# Activated blood-derived human primary T cells support replication of HAdV C5 and virus transmission to polarized human primary epithelial cells

**DOI:** 10.1128/jvi.01825-24

**Published:** 2025-04-23

**Authors:** Daniela Policarpo Sequeira, Maarit Suomalainen, Patrick C. Freitag, Andreas Plückthun, Michael Klingenbrunner, Lucy Fischer, Silvio Hemmi, Christian Münz, Romain Volle, Urs F. Greber

**Affiliations:** 1 Department of Molecular Life Sciences, University of Zürich 400018, Zürich, Switzerland; 2 Department of Biochemistry, University of Zürich 111970 , Zürich, Switzerland; 3 Institute of Experimental Immunology, University of Zürich 31010, Zürich, Switzerland; International Centre for Genetic Engineering and Biotechnology, Trieste, Italy

**Keywords:** adenovirus infection, primary human lymphocytes, persistent infection, primary human bronchial epithelial cells, air–liquid interface, DARPin, activated T cells, progeny virus production, protein adapter, E1A-2A-GFP expression

## Abstract

Human adenoviruses (HAdVs) cause self-limiting disease but are life-threatening to immunocompromised individuals. HAdV-C5 infects epithelial cells of the airways and eyes through aerosols, contaminated hands, or medical instruments, as well as fecal-oral contacts, gives rise to viremia, persisting in lymphoid cells of the gastrointestinal tract. Here, we show that pre-activated human primary blood-derived T cells can be infected with HAdV-C5 *in vitro*, upon incubation of the virus with a mixture of three distinct homotrimeric adapter proteins that target the virus to T cells. Each of the adapter proteins can bind 1 of the 12 fiber knobs of the virion through a designed ankyrin repeat protein. Two of the adapters contained a single-chain antibody fragment to T cell surface proteins CD3 or CD28, and the third one contained the cytokine interleukin-2. These adapters mediated efficient infection of primary T cells by HAdV-C5 and infectious progeny release, albeit with donor-to-donor variability. Co-culture of well-polarized air–liquid interface human bronchial epithelial cells with infected CD3^+^ T cells gave rise to progressively increased viral titers from replicating but not from replication-defective E1-deleted HAdV-C5, notably with similar kinetics as cell-free virus infections, suggesting that progeny virus from T cells was further amplified in epithelial cells. This study provides a platform to explore interactions between epithelial and immune cells in acute and persistent HAdV-C5 infection settings.

**IMPORTANCE:**

Many human adenoviruses (HAdV), including HAdV-C5, infect and propagate to high titers in epithelial cells of the airways. Virus ends up in lymphoid cells of the gastrointestinal and respiratory mucosa, where it can persist subclinically for years, restricted by memory T cells and humoral immune defense. In immunodeficient patients or newborns, however, HAdV can be fatal, coincident with lymphocytopenia and virus proliferation in epithelial cells. Here, we show that activated blood-derived human primary T lymphocytes can be productively infected with HAdV-C5 coated with trimerized adapter proteins targeting CD3, CD28, and the interleukin 2 receptors. A co-culture model of infected T cells and primary human bronchial epithelial cells in the absence of HAdV-specific immune cells showed that progeny virus from T cells was transferred to epithelial cells and led to increased progeny production compared to infected T cells alone, a situation potentially mimicking persistently infected mucosal lymphoid cells in immunosuppressed patients.

## INTRODUCTION

Viruses have a dual nature, passive and active. For the most part, virus particles (virions) and their enclosed genome are passive matter, while cells undergoing acute, persistent, or abortive infections are active matter. While acute infections often lead to viremia and abortive infections to elimination of viruses, persistence has frequently been considered a dead end to eukaryotic viruses owing to limited viral dissemination. However, persistent infections ensure the survival of viruses for extended periods of time (1), as in the case of human adenovirus (HAdV) (2, 3). Sometimes persistence is lifelong, as with herpesviruses (4–7) or retroviruses (8–10). Persistence occurs through complex and diverse mechanisms depending on the nature of the virus (11). In the case of DNA viruses, latency or persistence is linked to epigenetic silencing of viral gene expression. HIV-1 persistence and latency require viral genome integration into the host chromosomes, transition of the infected host T cell into a quiescent memory cell, and concomitant silencing of viral gene expression by host epigenetic mechanisms (12). Cell type-specific epigenetic silencing of viral gene expression is also the basis of herpesvirus latency (5, 6). An *in vitro* cell culture model system has suggested that interferon epigenetically curtails HAdV gene expression and allows viral persistence by continuous low-level virus replication and shedding under continued activation of the unfolded protein response sensor Ire1α (2, 3, 13–15).

HAdV was first isolated in adenoidal tissue in the 1950s (16, 17). HAdVs are nonenveloped double-stranded DNA viruses of the *Adenoviridae* family and the Mastadenovirus genus. They are transmitted by airborne, waterborne, fecal, oral, and fomite routes, as well as via contaminated medical instruments. The largely acid-resistant virus particles can persist in the environment (18–21). HAdVs are probably best known for their relatively mild, self-limiting infections, with clinical manifestations in the lower respiratory, digestive, and/or ocular tracts in immunocompetent individuals (for reviews, see references 22–25), besides being an effective vector in gene delivery (26, 27). However, HAdVs can also establish persistent infections. Persistent HAdV infections were first reported in the 1950s in lymphoid tissue of adenoids and tonsils of patients who underwent a tonsillectomy (28), particularly species C HAdV (3). Accordingly, viral DNA (vDNA) has been found in tonsil and adenoid tissues (29, 30). HAdV-C persistent infections are presumably a consequence of a viremic infection. They feature long-term, low-level shedding of progeny virus, causing little or no harm to host tissue (31–33). In fact, HAdV-Cs commonly infect the airways of young children and account for about 5%–7% of the respiratory tract infections in pediatric patients (34). Yet, how the virus disseminates from the respiratory tracts to mucosal tissues is unknown. Nonetheless, an important HAdV reservoir in children is in the gastrointestinal (GI) tract, namely intestinal lymphocytes embedded in the intestinal epithelium, from which the virus disseminates under immunosuppressive conditions, as shown by immunohistochemistry in biopsies from immunocompromised patients (2, 33, 35). This situation represents a life-threatening condition for immunocompromised patients, in particular, children undergoing hematopoietic stem-cell transplantation and exhibiting HAdV polymerase chain reaction (PCR)-positive stool probes (36–40). On an epidemiologic scale, the activation of persistent HAdV-C and intermittent shedding in the stool might maintain the virus in the population at large (34, 41).

The mechanisms that mediate the persistence of HAdVs in lymphocytes, as well as virus reactivation and spread of infection to epithelial cells, are poorly understood at the molecular level. This is, in part, due to a lack of primary cell infection models that recapitulate these events *in vitro*. In this study, we established an *in vitro* model for transferring HAdV-C5 infection from primary T cells to respiratory epithelial cells. Efficient entry of HAdV-C5 into T cells was achieved by coating purified virus particles with a mix of protein adapters that retarget HAdV-C5 to CD3, interleukin (IL)-2 receptors, and CD28 costimulatory receptors of primary human T lymphocytes (42). We show that pre-activated T cells are productively infected by the adapter-coated HAdV-C5. A co-culture model with human primary bronchial explant cells demonstrated that HAdV-C5 progeny derived from infected T cells is transmitted to epithelial cells, enabling virus amplification in the epithelial cells. These results provide a new model system to better understand HAdV-C5 infection and persistence in T lymphocytes and the underlying mechanisms of intra-host viral transmission.

## RESULTS

### A three-adapter protein strategy enables HAdV-C5 to infect activated primary T cells

HAdV-C5 is recognized as a prevalent type in lymphoid cells of the GI mucosa and stool samples, causing life-threatening viremia in immunosuppressed hematopoietic stem cell recipients, particularly children (37, 39, 43–45). Infection of primary human lymphoid cells with HAdV-C5 has been difficult to achieve *in vitro* (24, 46, 47). In the case of T cells, this is at least partially due to these cells expressing only low amounts of coxsackievirus adenovirus receptor (CAR) (48), the high-affinity surface receptor that mediates HAdV-C5 infection of epithelial cells (49).

**Fig 1:**
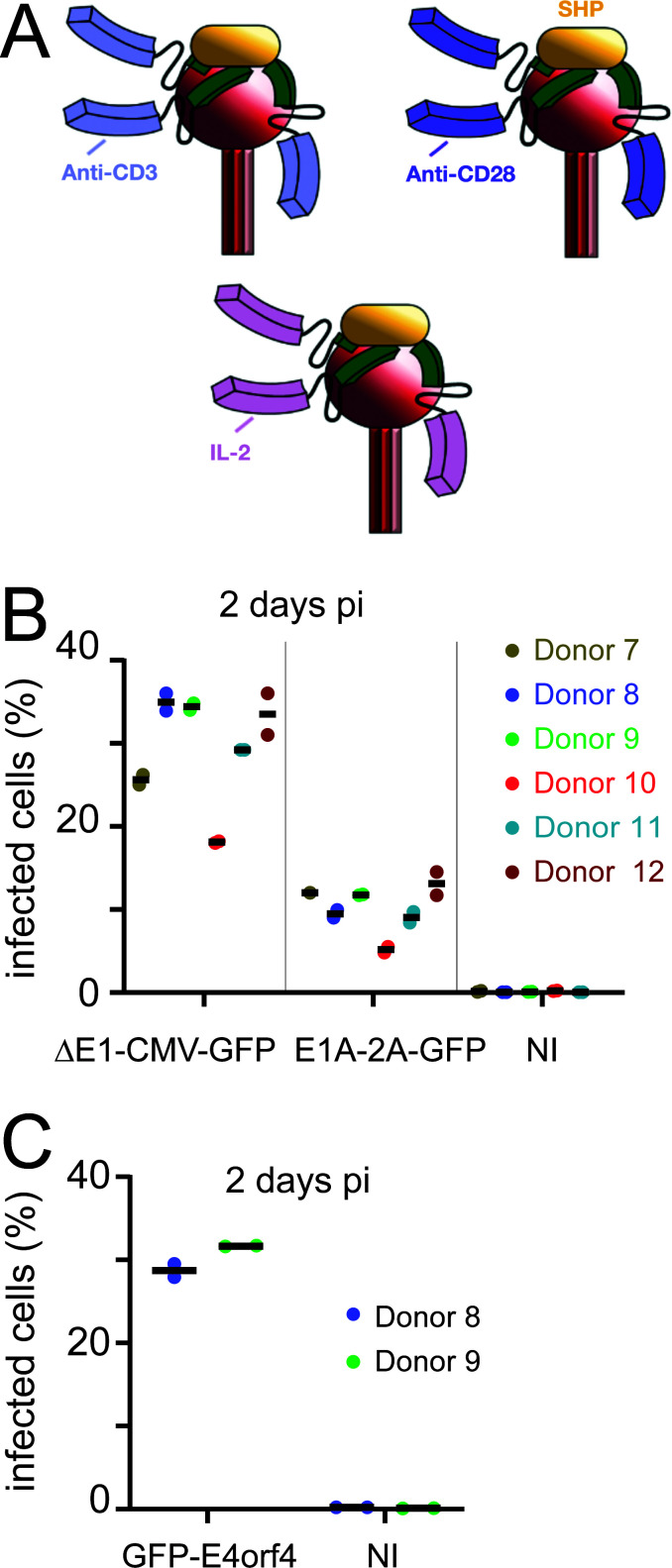
DARPin adapters enable infection of pre-activated human primary T cells. (A) Schematic depiction of the adapter complexes on the fiber knob. Adapters are homotrimers, and each of them binds a different fiber knob of the virion. IL-2 represents the natural interleukin-2 cytokine; anti-CD28 and anti-CD3 are scFv proteins derived from antibodies. They bind the IL-2, CD28, and CD3 receptors on the T cells. A DARPin (green) binds to the viral fiber knob (depicted as a red sphere), and a trimerization domain derived from the capsid-stabilizing protein (SHP) of the lambdoid phage 21 is shown in yellow. The fiber shaft trimer linking the knob to the viral capsid is shown as a stick in three shades of red below the knob. Adapted from reference 42. (**B**) Pre-activated primary T cells from six different donors were infected with adapter-coated replicating HAdV-C5-E1A-2A-GFP or non-replicating HAdV-C5-∆E1-CMV-GFP viruses. Non-infected (NI) cells were included as controls. Infection efficiencies, i.e., the percentage of GFP-positive cells, were scored at 2 dpi using flow cytometry. Shown are two technical replicates, with mean values for all other samples, except HAdV-C5-E1A-2A-GFP donor 7 infection reading was without a technical replicate, and non-infected controls for donor 12 were not included in the assay. Median GFP signals of the GFP-positive populations in the HAdV-C5-∆E1-CMV-GFP infections were between 1,206 and 2,471 and between 549 and 823 in the HAdV-C5-E1A-2A-GFP infections, whereas the median autofluorescence signals from the GFP-negative non-infected control cells were <35. (**C**) Flow cytometry analysis of infection efficiencies of HAdV-C5-GFP-E4orf4 in donor 8 and donor 9 pre-activated primary T cells at 2 dpi. The infection efficiencies are depicted by the percentage of GFP-positive cells. The two technical replicates are shown separately, along with their mean value. Median GFP signals of the GFP-positive populations in the infections were around 1,100, whereas the median autofluorescence signals from the GFP-negative non-infected control cells were <100.

A previous study described a three-adapter protein strategy to overcome the HAdV-C5 receptor shortage on primary T cells (42). One of the adapters binds to CD3, one to CD28, and the third one to the IL-2 receptor (see Fig. 1A and reference 42). Each adapter consists of one designed ankyrin repeat protein (DARPin), which binds to the AdV fiber knob, one trimerization domain from the phage protein SHP, plus a single chain variable fragment (scFv) from either antibodies against CD3 or CD28, or the natural ligand IL-2 (50–52). Targeting with the CD3, CD28, and IL-2 receptors simultaneously attempts to mimic an *in vivo* immune synapse (53), a state triggered by the engagement of the T cell receptor with viral antigens presented by MHC on antigen-presenting cells (APCs) (reviewed in reference 54). In addition to the adapters, an efficient transduction of primary T cells by a replication-deficient HAdV-C5 vector also required preactivation of the cells with anti-CD3 and anti-CD28 antibodies (42).

In the present report, we tested whether the above-described adapters would be good tools for studying the full HAdV-C5 replication cycle in primary T cells. We used different types of replicating HAdV-C5 reporter viruses in the experiments described below. Our replicating HAdV-C5-E1A-2A-GFP virus directs the synthesis of a hybrid transcript from the native AdV E1A enhancer/promoter (e/p), which yields both E1A and green fluorescent protein (GFP) proteins through ribosomal skipping during translation (55). This virus has slightly slower growth kinetics in A549 cells than wild-type (wt) HAdV-C5 virus (multiplicity of infection, MOI 1) but reaches similar endpoint titers as the wt virus (55). HAdV-C5-IX-2A-GFP is a virus that replicates as efficiently as wt HAdV-C5 in A549 cells and expresses the intermediate-late viral protein IX and GFP from a shared mRNA via ribosomal skipping (55). In the replicating HAdV-C5-GFP-E4orf4, the early protein E4orf4 is genetically fused to GFP (56). This virus replicates more slowly than wt HAdV-C5 in A549 cells (MOI 1 infection) but reaches titers that are only about threefold lower than those of the wt virus at 80 h post-infection (pi) (M. Suomalainen, unpublished results). We also used a non-replicating HAdV-C5-∆E1-CMV-GFP as a control virus. Non-replicating HAdV vectors can be generated by deleting the viral early E1 transcription units, and the HAdV-C5-∆E1-CMV-GFP carries a GFP reporter sequence under the control of a cytomegalovirus (CMV) immediate early e/p in place of the E1 units (57).

Figure 1B shows flow cytometry analyses of adapter-coated HAdV-C5-∆E1-CMV-GFP infection in pre-activated primary CD3^+^ T cells, which were derived from six independent donors (donors 7–12). The addition of adapters to HAdV-C5-∆E1-CMV-GFP enabled efficient transduction of the cells with donor-dependent efficiencies between 18% and 36% at 2 days pi (dpi), akin to previous results (42). Similarly, up to about 15% infection efficiencies were achieved with the adapter-coated replicating HAdV-C5-E1A-2A-GFP (Fig. 1B). The dissimilar infection efficiencies of HAdV-C5-∆E1-CMV-GFP and HAdV-C5-E1A-2A-GFP most likely reflect differences in the promoter activities driving the GFP expression (CMV immediate early vs HAdV E1A promoter). The assay was repeated in donors 8 and 9 with the replicating HAdV-C5-GFP-E4orf4 virus, and GFP signal was observed in 28%–32% of the cells at 2 dpi. Taken together, these results indicate that the adapter proteins enable efficient entry of HAdV-C5 viruses into the T cells and the start of virus gene expression. All experiments described below used adapter-coated viruses and pre-activated T cells.

### HAdV-C5 replicates in blood-derived T lymphocytes and releases infectious progeny

**Fig 2:**
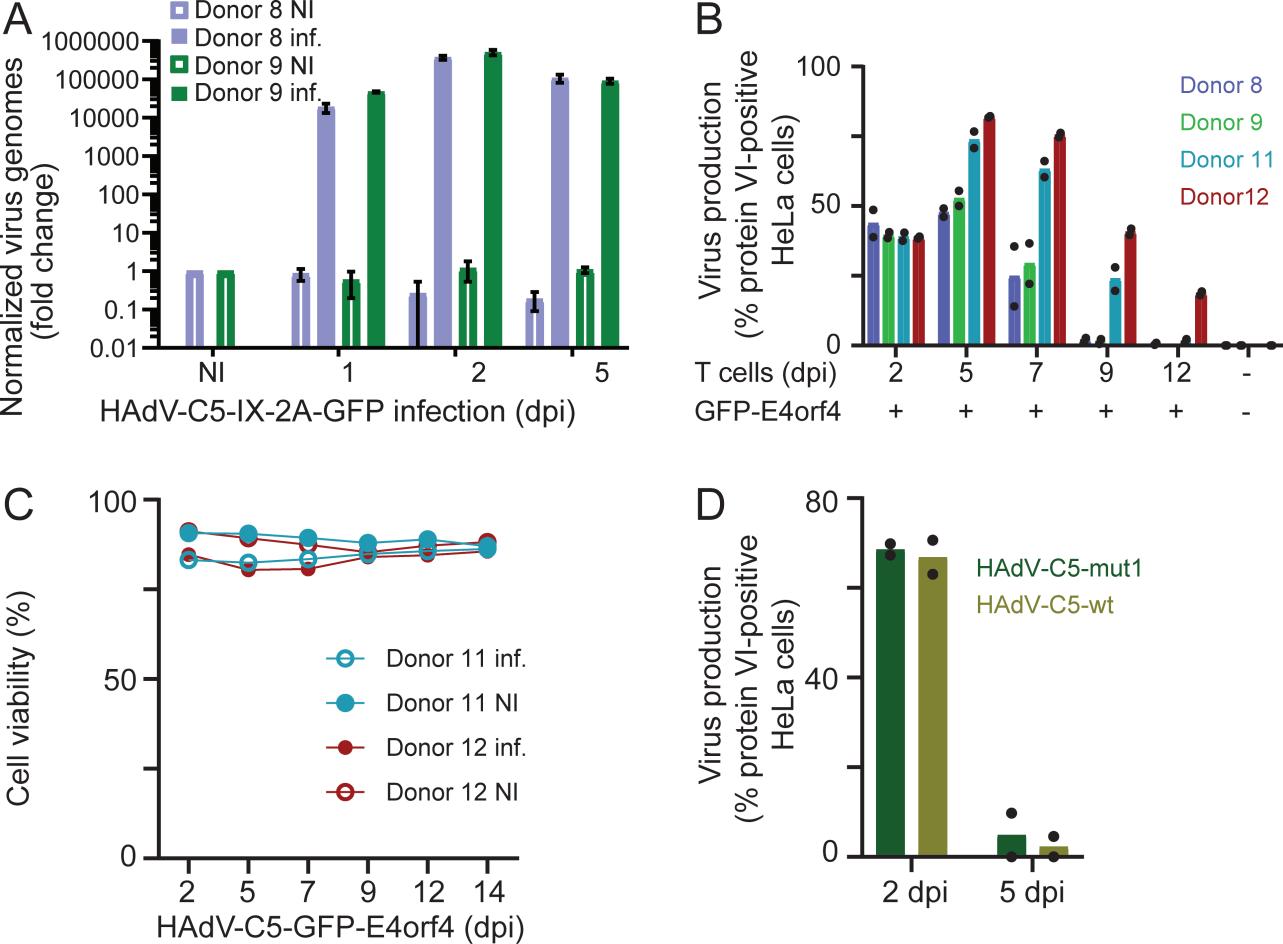
HAdV-C5 coated with DARPin adapters replicates and produces progeny in the pre-activated primary T cells. (**A**) After infection (inf) of pre-activated primary T cells with adapter-coated HAdV-C5-IX-2A-GFP, DNA was extracted at 1, 2, and 5 dpi, and qPCR was performed using primers targeting E1A promoter, and host MARCO gene for normalization. Negative controls with non-infected (NI) samples were included for all time points. vDNA copy numbers (shown as means of three technical replicates with SD) were estimated from standard curves for E1A and MARCO and normalized to NI samples collected prior to infection. (**B**) To probe for kinetics of progeny production from infected T cells, pre-activated T cells from donors 8, 9, 11, and 12 were infected with adapter-coated HAdV-C5-GFP-E4orf4, and clarified culture supernatants were collected at 2, 5, 7, 9, and 12 dpi and titrated on HeLa cells. Infected cells were visualized at 42 hpi by immunofluorescence using antibodies against the viral late protein VI and an AlexaFluor 594-conjugated secondary antibody. Nuclei were stained with DAPI. The number of infected cells was scored by fluorescence microscopy. Black circles correspond to T cell supernatant technical replicates. (**C**) Cell viability of pre-activated donor 11 and 12 cultures infected with adapter-coated HAdV-C5-GFP-E4orf4 was analyzed on the CASY cell counter. NI samples were used as controls. (**D**) Interferon (IFN)-mediated repression of the virus E1A enhancer/promoter (e/p) does not appear to be a major or the only regulator of T cell infection with HAdV-C5. Pre-activated donor 9 T cells were infected with adapter-coated HAdV-C5-wt or HAdV-C5 e/p mutant virus (Mut1). Mut1 is largely insensitive to IFN-mediated repression of the E1A e/p activity. Clarified culture supernatants were collected at 2 and 5 dpi and titrated on HeLa cells. HeLa infection efficiencies were scored at 42 hpi by immunostaining for protein VI. Fluorescence microscopy was used for determining the number of VI-positive cells. The bars represent mean values from two technical replicates, with the individual replicate values shown by black circles.

We next investigated whether viral genome replication could be observed in the primary CD3^+^ T cells. Activated CD3^+^ T cells from donors 8 and 9 were infected with adapter-coated HAdV-C5-IX-2A-GFP. Whole T-lymphocyte DNA was extracted from the cells at different times post-infection and processed for quantitative PCR (qPCR) analysis of viral genomes using E1 promoter target-amplification. T cell number normalization was performed using scavenger receptor SR-A6 (MARCO)-targeting primers (Fig. 2A). The values from 1 dpi represent input cell-associated vDNA. The data in Fig. 2A show that vDNA replication in both donors increased until 2 dpi, with about 20- and 11-fold increase in normalized vDNA copy numbers between day 1 and day 2 pi in cells from donor 8 and donor 9, respectively. Overall, the replication was rather inefficient. For comparison, a 1,000-fold increase in infectious progeny (pfu/cell) has been reported for HAdV-C5-IX-2A-GFP infection in human lung carcinoma A549 cells by 2 dpi at initial MOI 1 infection (55). At 5 dpi, vDNA copy numbers were still elevated in the infected T cells in comparison to 1 dpi but not in comparison to 2 dpi, giving rise to the possibility that a small degree of cell death might have occurred between these two time points.

To explore if infected primary human CD3^+^ T cells not only replicated vDNA but also released infectious HAdV-C5 progeny, we collected clarified culture supernatants of HAdV-C5-GFP-E4orf4-infected T cells from donors 8, 9, 11, and 12 and used these culture supernatants to infect HeLa cells. HeLa cells were stained for the late viral protein VI 42 hpi. Of note, possible carryover of contaminating adapter-coated virus from the original T cell infection would not initiate infection in HeLa cells since adapter-coated viruses are impaired in the interaction with the HAdV-C5 receptor CAR (51). While donor 8 and 9 T cells released progeny for up to 7 dpi, donor 11 cells released viral particles up to 9 dpi and donor 12 cells up to 12 dpi (Fig. 2B). We also monitored cell viability in the HAdV-C5-GFP-E4orf4-infected donor 11 and 12 cultures and observed essentially no impact of infection on the overall culture viabilities (Fig. 2C).

Although this study does not address the molecular mechanisms of HAdV-C5 infection in primary T cells, we tested if the type I and II interferon (IFN)-insensitive HAdV-C5 mutant gave rise to increased progeny from the infected T cells. The HAdV immediate early E1A proteins are essential for efficient activation of viral transcription units and restructuring of host gene expression to implement a cell state favorable for productive virus replication (58–62). In IFN-treated cells, an E2F transcription factor binding site on the E1A enhancer mediates the recruitment of Rb-containing repressor complexes to the E1A e/p, thus leading to a low level of E1A expression and inefficient virus replication (13, 14). HAdV-C5-mutant 1 (mut1) contains 12 clustered point mutations in this E2F binding site of the E1A e/p, and this renders these viruses less susceptible to IFN-mediated replicative suppression (13). Infection of preactivated T cells from donor 9 with mut1 did not result in increased virus progeny production in comparison to HAdV-C5 wt infection (Fig. 2D). These data suggest that repression of E1A e/p by autocrine-produced IFN-I or -II is not a major restriction point of HAdV-C5 infection in primary T cells or, alternatively, is not the only restriction point.

### Infected CD3^+^ T cells spread HAdV-C5 progeny to primary bronchial epithelial explant cultures

**Fig 3:**
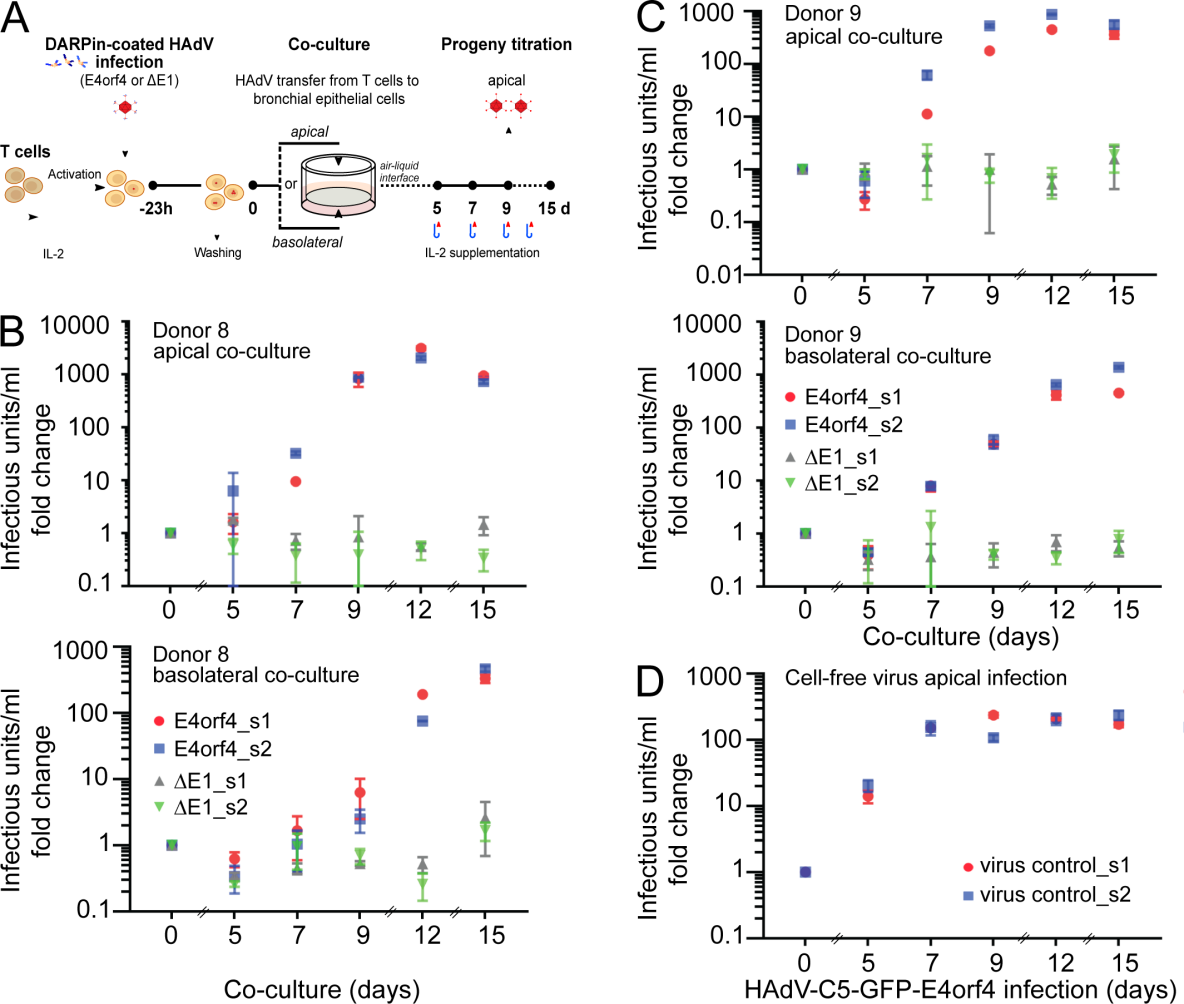
Bronchial epithelial cells are infected with T cell-derived progeny. (A) Schematic procedure for co-culturing bronchial epithelial cells with HAdV-C5-infected T cells. Preactivated T cells from two different donors, donor 8 (B) and donor 9 (C), were inoculated with DARPin adapter-coated HAdV-C5-GFP-E4orf4 (E4orf4) or HAdV-C5-∆E1-CMV-GFP (∆E1). After 23 h, T cells were extensively washed to remove unbound virus. Supernatant from the last wash was collected, and it refers to day 0 virus in the T cell–ALI co-culture progeny titrations in panels B and C. Infected T cells were added to the basolateral or apical side of polarized bronchial human airway epithelial ALI cultures. Two technical replicates (s1 and s2) were prepared for each sample. T cells were incubated with the ALI cultures at 37°C for 3 h. Control inserts were infected with free HAdV-C5-GFP-E4orf4 virus from the apical side (D). After the 3-hour virus incubation, ALI culture plates were centrifuged to allow the T cells to settle, and supernatants from both sides were discarded. The apical side of the control wells with the cell-free virus infection was washed twice with medium, the last wash was collected, and it refers to the day 0 free virus in the progeny titrations. The ALI cultures were incubated at 37°C in ALI culture medium containing 50 units/mL IL-2 added to the basolateral side, and the inserts were transferred to a fresh medium every 2–3 days. Beginning on day 5 post-T cell addition, apical washes were collected at 2–3-day intervals for virus progeny titrations. The progeny titers were determined on HeLa cells by scoring for GFP-positive cells at 24 hpi by fluorescence microscopy. Virus progeny titers were estimated from wells that displayed 20%–40% infection efficiencies, and one GFP-positive cell was taken as one infectious unit. The infectious titers were normalized to the day 0 values. Shown are mean values ± SD from three technical replicates.

Co-culture of immune cells and epithelial cells mimics an *in vivo* infection scenario closer to *in vivo* airways than distinct cell types alone. We aimed to investigate whether progeny HAdV-C5 released from T cells was able to infect primary epithelial cells. We compared the kinetics of infection from the basal and apical sides, in both co-culture and air–liquid interface (ALI) monoculture. The experimental setup is schematically shown in Fig. 3A. Pre-activated T cells from donors 8 and 9 were infected with adapter-coated HAdV-C5-GFP-E4orf4 or HAdV-C5-∆E1-CMV-GFP for 23 h. Free virus was removed by extensive washing, and T cells were added to well-polarized bronchial human airway epithelial cells (ALI cultures) either at the air-exposed apical side or the media-exposed basolateral side for 3 h. Control ALI cultures were apically inoculated with cell-free virus lacking the adapters. Of note, in this setup, low levels of contaminating free adapter-coated virus from T cells would not yield ALI-culture infection since adapter-coated viruses are impaired in the interaction with the HAdV-C5 receptor CAR (51). We found that after the removal of the inoculation medium, apically added T cells remained attached to the apical side of the polarized epithelial cells, whereas basolaterally added T cells settled to the bottom of the well. Microscopy analyses of the ALI cultures indicated GFP-positive epithelial cells at day 5 post-inoculation with T cells infected with adapter-coated HAdV-C5-GFP-E4orf4. From this time point onward, apical washes were collected at 2- or 3-day intervals, and virus titers in the apical washes were determined by titration on HeLa cells, using input dilutions that resulted in about 20%–40% infection efficiencies. Accordingly, one GFP-positive HeLa cell was taken as one infectious unit. The infectious titers on HeLa cells were normalized to day 0 values, which refer to the supernatant from the last wash of the infected T cells prior to the addition of the T cells to the ALI cultures.

T cell-mediated transfer of HAdV-C5-GFP-E4orf4 infection to ALI cultures was successful both from the apical and basolateral side, with apical T cell co-culture yielding a more rapid rise of virus progeny. Virus progeny reached peak levels at 12 days post-apical co-culturing and at 15 days post-basolateral co-culturing (Fig. 3B and C). This was much delayed compared to virus production in T cells, which peaked at 5–7 dpi (Fig. 2B), strongly suggesting that virus progeny did not represent virus released from T cells but was derived from the epithelial cells. In contrast, progeny production from ALI cultures infected with cell-free virus peaked at 7 dpi (Fig. 3D). Progeny titers from cell-free virus infections and T cell-mediated infections were similar at peak levels, between 4.5 and 14 million infectious units per milliliter (data not shown). The highest values originated from donor 8 T cell-mediated basolateral and donor 9 T cell-mediated apical infections. Interestingly, production kinetics were faster with apical than basolateral T cell inoculation (Fig. 3B and C), possibly due to close contacts of T cells with apical epithelial cells. Infection from the basolateral side could have been at least partially hampered by the insert membrane. Importantly, the infected ALI cultures remained apparently intact until at least day 15 pi, as indicated by the lack of culture medium on the apical side as judged by microscopic inspection. However, we acknowledge that medium leakage to the apical side is only an approximate measure of the intactness of tight junctions and does not exclude the possibility that the infection caused small local changes in the permeability of the polarized cells.

Collectively, the data show that primary bronchial epithelial cells were infected with T-cell-derived viral progeny at both the apical and basal compartments, yielding virus progeny from ALI-T cell co-cultures similarly as with cell-free virus infections. This co-culture system hence provides a feasible *in vitro* approach to study interactions of immune cells with epithelial cells in the context of HAdV infection.

## DISCUSSION

T cells represent cell types that can harbor persistent HAdV, and species C HAdVs are commonly detected viruses in persistent HAdV infections (for example, references 32, 33). HAdV-C persistence in T cells has mainly been studied in cell lines as opposed to primary cells, owing to the scarcity of a high-affinity binding site on primary T lymphocytes for HAdV-C and low levels of RGD-binding integrins (48, 63, 64), both of which render human epithelial cells susceptible to HAdV-C infection (24). Here, we overcome these receptor limitations by retargeting HAdV-C5 to T cells using highly specific DARPin adapters, which bridge the virus to different T-cell receptors and also activate the T cells, thereby enhancing transduction and infection efficiency *in vitro* and *in vivo* (this study and reference 42). Of note, pre-activation of T cells by anti-CD28, anti-CD3, and recombinant IL-2 is necessary for an efficient infection with HAdV-C5 (42). This resonates with earlier reports showing that persistent (latent) HAdV-C is present in tonsils and adenoids (28, 30, 65), preferentially in quiescent T-lymphocytes (29), and virus replication from tonsillar tissue could be reactivated by *in vitro* stimuli (66).

The available HAdV persistence models are derived from transformed T lymphocytes, which can be infected with HAdV-C5 despite low levels of CAR expression, yielding late viral proteins, such as hexon, for several months without compromising cell proliferation and survival (63, 67). In our present study with pre-activated blood-derived primary T cells infected with HAdV-C5, the overall viability of the infected cultures up to 14 dpi was not compromised. We observed detectable early shedding of infectious virus up to 12 dpi, though with notable donor-to-donor variability. Reasons for this rather short-term HAdV “persistence” in our primary T cell infections could be several fold. One possibility is that the nuclear viral genomes became epigenetically silenced. Another possibility is that the initially infected cells proceeded to lytic infection, but without the retargeting DARPin adapters, second-round infections did not occur. In the presence of a proliferating non-infected cell fraction (42), this would lead to a time-dependent decline in progeny virus yields. Low levels of infectious progeny production and subsequent low levels of re-infections are most likely the mechanisms that maintain HAdV-C persistence in IFN-treated immortalized human dermal fibroblasts despite the actively cycling phenotype of these cells (13, 15). Overall, the relatively efficient infection of T cells by our adapter-coated HAdV-C5 opens up new research avenues for virus-host interactions in these cells. For example, the mechanism of progeny release from infected T cells in lytic and non-lytic modes (68, 69) could be addressed in future experiments by fluorescence-activated cell sorting of GFP-expressing infected cells and long-term follow-up studies.

Infection of the pre-activated T cells with the adapter-coated HAdV-C5 led to virus genome replication and a full virus infection cycle, resulting in the release of progeny to the culture medium. The replication and progeny release, however, occurred only at a relatively low level compared to human cancer cells. Estimating from the cumulative progeny yield from 2 to 12 dpi (Fig. 2B) and taking into account that only a fraction of the activated target T cells initiated HAdV-C5-GFP-E4orf4 infection (Fig. 1C), donor 11 cells released <100 infectious units per infected cell during this time period. Since we did not measure possible cell-associated progeny in the experiment in Fig. 2B, this estimation could be less than the actual total progeny yield. It is also possible that the infectious virus progeny in the culture supernatants originated from only a fraction of the infected cells, reflecting cell-cell infection variability (70, 71). We speculate that the rather modest virus replication in the T cells may have contributed to the extended viability of the infected lymphocytes. The relatively high transduction efficiencies achieved with the adapter-coated HAdV-C5-∆E1-CMV-GFP virus suggest that virus entry into the cells occurred efficiently. Thus, our system offers a platform to study the nuclear phase of HAdV-C infection in primary T cells and the obstacles these cells impose on virus amplification.

When co-cultured with human bronchial cells, the infected primary T cells passed the virus onto epithelial cells, which in turn amplified the agent. The co-culture model of T lymphocytes and bronchial epithelial cells was inspired by the *in vivo* observation that immune cells populate the epithelial basal side of the mucosa, whereas ciliated cells reside on the apical side of the polarized epithelium (72), where viruses get access to the mucosa and may be transmitted to the immune cells. Interestingly, our results show that polarized primary bronchial epithelial cells could be infected by apical inoculation of T cells harboring replicating HAdV. This is in contrast to cell-free virus inoculation of polarized 16HBE14o cells (an SV40-immortalized human bronchial epithelial cell line), which required the addition of recombinant IL-8 or co-culture with IL-8-producing macrophages for infection (73). Possibly, this difference also relates to the observation that the CAR^Ex8^ isoform of the HAdV-C high-affinity CAR receptor can localize at the apical epithelial cell surface, where it can play a role in lumenal AdV infection (74). Interestingly, IL-8 has been shown to increase CAR^Ex8^ expression at the apical surface of polarized primary human airway tracheal epithelial cells (75). Future experiments might test if IL-8 could boost the T cell-mediated HAdV-C apical infection transfer to epithelial cells. Furthermore, the effects of infected T cells on the HAdV infection phenotype in epithelial cells are an interesting topic for future research. For example, human nasal epithelial cells apically infected with rhinovirus 14 and co-cultured with activated T cells revealed that the T cells modify the antiviral and anti-inflammatory response of the epithelial cells by triggering overexpression of the pro-inflammatory chemokine CXCL10 (76).

In summary, we provide a new model for HAdV-C5 infection in human primary T lymphocytes, showing that pre-activated T cells carried out productive HAdV-C5 infection. With recent advances in siRNA- or CRISPR-Cas9-mediated gene silencing in primary T cells (77, 78), our system may now be expanded toward molecular characterization of HAdV-host interactions in this cell type. The co-culture of infected T cells with human bronchial epithelial cells offers the possibility to conduct drug screenings that mimic preemptive and post-infection treatments and immunosuppression scenarios. It also allows for further studies of the infection dynamics in immune-epithelial cell mixed cultures and the impact of immunosuppression. We also envision that our T cell infection model could be combined with gut epithelial cultures, possibly using induced pluripotent stem cells as a source for both T cells and gut epithelium (79, 80). However, we acknowledge that our system has limitations as well. For example, efficient HAdV-C5 infection of activated T cells was achieved only with synthetic adapter proteins, and this limits the study of virus infection in these cells to a single-round infection. Furthermore, although our setup can reproduce T cell-to-epithelial infection transfer, studies of the reverse scenario, that is, how T cells get infected in mucosal tissues in the absence of synthetic adapters, might be difficult to achieve in our coculture ALI system. This can be challenging unless the T cells carry accessible markers for cell type identification and are assessed for infection status by early or late viral gene expression reporters.

## MATERIALS AND METHODS

### Cell lines and viruses

Cell lines were grown in Dulbecco’s Modified Eagle’s Medium (DMEM, Gibco) at 37°C and 5% CO_2_ humid atmosphere conditions, supplemented with 1% (vol/vol) non-essential amino acids (NA, Thermo Fisher), 10% (vol/vol) heat inactivated fetal calf serum (FCS), 1% (vol/vol) penicillin-streptomycin (PenStrep) (termed complete DMEM). HeLa (human epithelial cervix carcinoma; ATCC CCL-2) and A549 (human adenocarcinomic alveolar basal epithelium; ATCC CCL-185) cells were obtained from the American Type Culture Collection (ATCC) (USA). Cells were routinely passaged following phosphate-buffered saline (PBS) washing and subsequent trypsinization. The HER-911 cells (human embryonic retinoblasts) were a kind gift from Robert Hoeben, Leiden University Medical Center, the Netherlands (81).

HAdV-C5 wild type, HAdV-C5-E1A-2A-GFP, and HAdV-C5-IX-2A-GFP viruses were propagated in A549 cells. The non-replicating HAdV-C5-ΔE1-CMV-GFP vector (57), an E1/E3 deletion mutant virus expressing enhanced green fluorescent protein (GFP) from a cytomegalovirus major immediate early enhancer/promoter, was grown in HER-911 cells. The replicating HAdV-C5-GFP-E4orf4 virus expresses a GFP-E4orf4 fusion protein (56) and was grown in A549 cells. In the replicating HAdV-C5-mut1 virus, one of the E2F binding sites on the E1A enhancer has been mutated (13). This virus was a kind gift from Patrick Hearing, Stony Brook University, USA, and the virus was grown in HEK-293 cells (ATCC, CRL-1573). The replicating HAdV-C5-E1A-2A-GFP virus encodes both E1A and GFP from the native E1A enhancer/promoter, and the two coding sequences are separated by a ribosome-skipping 2A sequence from foot and mouth disease virus (55, 82). In the replicating HAdV-C5-IX-2A-GFP virus, the 2A sequence mediates both virus protein IX and GFP expression from a common mRNA transcribed from the native IX promoter (55). HAdV-C5-E1A-2A-GFP and HAdV-C5-IX-2A-GFP viruses were propagated in A549 cells. Progeny viruses were purified on two consecutive cesium chloride gradients as previously described (83), and aliquots were stored at −80°C.

### Cultivation and activation of blood-derived T lymphocytes

Buffy coats obtained from Blutspende SRK Zurich, Switzerland, were used for the isolation of peripheral blood mononuclear cells (PBMCs) using Ficoll (GE17-1440-02) gradient centrifugation. PBMCs were frozen at −80°C in 90% cell culture medium and 10% DMSO and kept in liquid nitrogen for long-term storage. Cells were cultivated in suspension at 37°C and 5% CO_2_ humid atmosphere conditions in complete RPMI medium (RPMI Sigma 8758, 10% heat-inactivated [vol/vol] FCS, and 1% [vol/vol] PenStrep) and counted using a CASY cell counter (Model TT, Innovatis, OMNI Life Science). Medium was replenished every 2–3 days with 50 U/mL of IL-2 (Peprotech 200-02) added for cell maintenance. For chemical activation and purification of T lymphocytes from PBMC suspensions, cells were thawed and immediately seeded at a concentration of 1 × 10^6^ cells/mL in complete RPMI (see above) and supplemented with 200 U/mL of IL-2. Dynabeads (Thermo Fisher 11131D) covalently coupled to anti-CD3 and anti-CD28 antibodies were added to the PBMC suspension with a bead/cell ratio of 1 for 24 h in tissue culture flasks (25 or 75 cm^2^). After 24 h of activation, cells were centrifuged at 350 × *g* for 5 min, resuspended in 1 mL of complete RPMI, and separated from the CD3-negative population with an EasySep magnet (Stemcell Technologies 18000). Cells were immediately used for infection as described below.

### Infection assays of primary T lymphocytes

Fifty thousand pre-activated T lymphocytes were seeded per well of 96-well round-bottom plates and immediately infected by inoculating a mix of HAdV-C5 and three adapter proteins designed for HAdV-C5 vector transductions as previously described (42). Briefly, the designed ankyrin repeat protein adapter proteins used contained human IL-2 as a ligand for IL-2R or scFv targeting the T lymphocyte ubiquitously expressed membrane proteins CD3 or CD28. The molar ratio of each adapter to fiber knob was 10.8 to 1 in the coating reaction (see Fig. 1A). Adapter proteins bind to the HAdV-C5 fiber knob by virtue of a DARPin (52). Incubation of adapters/virus was conducted for 1 h on ice, after which the mixture was diluted with complete RPMI and added to the T cells. A total multiplicity of infection of 20,000 (virus particles/cell) was used. After an overnight inoculation at 37°C, cells were washed twice with complete RPMI media by centrifugation at 350 × *g* for 5 min and cultivated in the absence of IL-2 for 48 h. For a long-term culture, the medium of the infected T cells was replaced by fresh medium containing 50 U/mL of IL-2 every 2–3 days, and culture viability was followed by counting the number of viable cells at different times pi using a CASY cell counter.

### Flow cytometry

T cells were centrifuged at 750 × *g* for 5 min and resuspended in FACS buffer (PBS, 2% [vol/vol] heat-inactivated FCS, and 0.5 mM EDTA) for staining with fluorescently labeled antibodies against cell surface markers. After washing unbound antibodies away with FACS buffer, cells were fixed with 3% paraformaldehyde (PFA)/PBS for 20 min at room temperature and washed once with FACS buffer. Analysis was done with a 96-V-bottom plate using BD FACSymphony A5 high-throughput cell analyzer (BD Biosciences). However, we report here only analyses of the GFP signal, the marker for infected cells, since the source data for the single-stained compensation samples could not be recovered or the compensation sample data were corrupted. The GFP signal was not significantly affected by the other antibodies and could be analyzed without a compensation matrix since the antibodies used were conjugated to BV785, Alexa Fluor 700, PerCp-Cy5.5, PE, APC, BV605, PE-Cy7, or Alexa Fluor 594, none of which has a significant signal leakage to the GFP-recording Blue 530/30 channel in FACSymphony A5. Data analysis was performed with FlowJo software. Unstained non-infected control cells were used to set the gating in FSC-A vs SCC-A and FSC-A vs FSC-H parameters, as well as establishing the GFP fluorescence threshold for an infected cell. GraphPad Prism (GraphPad Software, La Jolla, CA, USA) was used for visual representation of the data.

### Quantitative PCR analysis

DNA was extracted from cells using the DNeasy Blood & Tissue kit (Qiagen) following the supplier’s protocol. Specific primers targeting the HAdV-C5 E1A promoter were used to detect viral genomes (E1A_Fw 5′-GGTGGAGTTTGTGACGTGG-3′ and E1A_RV 5′-CGCGCGAAAATTGTCACTTC-3′) (15), and MARCO-specific primers were used for cellular genome normalizations (MARCO_Fw 5′-TCAATGACACTCTGGCGGC-3′ and MARCO_RV 5′-CAAGTGCTCATGGCTGACG-3′). Plasmids containing E1A cDNA or MARCO cDNA were used to establish standard curves. qPCR was carried out with SYBR Green JumpStart TaqReadyMix (Sigma-Aldrich S4438), 10 µM forward/reverse primers, and pretested amounts of DNA samples using MicroAmp Optical 96-well Plates (Applied Biosystems N8010560) with Axygen UC-500 sealing films and QuantStudio 3 Real-Time PCR System (Applied Biosystems) under the following conditions: initial denaturation at 95°C for 10 min, 40 cycles of denaturation at 95°C for 30 seconds, and annealing and extension at 58°C for 1 min. Melt curve settings were 15 seconds at 95°C, 1 min at 60°C, and 1 second at 95°C (continuous collection mode). Results were analyzed using the QuantiStudio Design and Analysis Software version 1.5.1. GraphPad Prism was used for visual representation of the data.

### Progeny production from infected T lymphocytes

Supernatant from HAdV-C5-infected T lymphocytes was cleared from cells by centrifugation at 350 × *g* for 5 min. Samples were then twofold diluted in complete DMEM and inoculated onto HeLa cells, which had been seeded on a 96-well imaging plate (Greiner Bio-One, 655090) 1 day prior to infection at a cell density of 10,000 cells/well. Following inoculation with the T lymphocyte supernatant, HeLa cells were incubated for 42 h, fixed with 3% PFA/PBS for 20 min at room temperature, washed twice with PBS, quenched with 25 mM NH_4_Cl in PBS for 10 min, and permeabilized with 0.5% Triton X-100 in PBS for 5 min. Staining was performed with a rabbit anti-protein VI primary antibody for 1 h at 4°C (84) diluted 1:2,000 in blocking buffer (10% goat serum in PBS). Cells were washed three times with PBS and incubated for 30 min at RT with a goat anti-rabbit AlexaFluor 594 (Thermo Fisher Scientific A-11012, final concentration 2 µg/mL) secondary antibody diluted in blocking buffer supplemented with 1 µg/mL DAPI. Following secondary antibody incubation, cells were washed three times with PBS. Imaging was carried out in PBS using Molecular Devices high-throughput microscope (IXM-C) in widefield mode with a 10× Plan Apo Lambda objective.

### Air–liquid interface cell differentiation and cultivation

Human Airway Epithelial cells (62-year-old Hispanic male donor, non-smoker) were from a bronchial biopsy and obtained from Epithelix (product code EP51AB, batch number 02AB0793.01). Quality control screening for mycoplasma and virus contamination was performed by the supplier. Passage one undifferentiated cells were bought from the supplier, and differentiation was done in-house following guidelines from the commercial manufacturers Stemcell Technologies and Epithelix.

After thawing, cells (about 330,000) were taken into Stemcell Pneumacult Ex Medium (05008 with 50× supplement added to 1×) supplemented with hydrocortisone solution (Stemcell Technologies 07925, 1/1,000 dilution), and cells were allowed to reach 80%–90% confluence in a T-25 flask at 37°C, 5% CO_2_, as described (85). Next, cells were transferred to 6.5 mm 24-well inserts with a pore size of 0.4 µM (CLS3470-48EA, Corning) that had been coated with 50 µL of 0.25 mg/mL human placental collagen Type IV solution (C5533, Sigma/Merck) overnight at room temperature. After removing the coating liquid, inserts were treated with UV for 30 min and rinsed twice with 250 µL PBS. A total of 0.5 × 10^5^ cells were added to each collagen-coated insert, and inserts were incubated in liquid–liquid interface on average for 4 days in Stemcell PneumaCult EX Medium supplemented with added hydrocortisone. After cells had reached confluency, they were switched to air–liquid culture, and media on the basolateral side were replaced by Stemcell PneumaCult ALI medium (Stemcell Technologies 05001) supplemented with 0.48 µg/mL hydrocortisone, 4 µg/mL heparin (Stemcell Technologies 07980), and 150 ng/mL retinoic acid (Sigma R2625). Cultures were incubated for 4–6 weeks to allow differentiation and visually inspected under a microscope to look for mechanical beating of the ciliated cells. Media were changed every 2–3 days, and an apical wash was made every 4 weeks to remove mucus.

### ALI co-culture with T cells and infection assay

To quantitatively assess the ability of infectious T cell progeny to productively infect the ALI cultures, preactivated T cells from two different donors (donors 8 and 9) were infected with DARPin adapter-coated HAdV-C5-GFP-E4orf4 or HAdV-C5-∆E1-CMV-GFP for 23 h as described above. Afterward, T cells were washed three times with culture medium to remove the remaining free virus (centrifugation at 350 × *g* for 5 min to pellet the cells between washes). The supernatant from the last wash was collected, and it refers to the day 0 virus in the T cell–ALI co-culture progeny titrations (see below). Infected T cells (200,000 cells/insert) were added to the basolateral or apical side of ALI cultures in ALI culture medium containing 50 units/mL IL-2. The medium volume was 200 µL on the apical side and 500 µL on the basolateral side. Two technical replicates (samples 1 and 2) were prepared for each sample. Prior to the T cell addition, the apical side of the ALI cultures was washed with 200 µL ALI medium for 20 min at 37°C to reduce the amount of mucus. T cells were incubated with the ALI cultures for 3 h at 37°C. Control inserts were infected with free HAdV-C5-GFP-E4orf4 virus from the apical side. The estimated MOI for this control was 720 virus particles/cell. The MOI was calculated by taking into account that the cell growth area of the CLS3470-48EA insert is 0.33 cm^2^ and fully differentiated ALI cultures have around 3 × 10^6^ cells/cm^2^ (86). After the 3-hour incubation, ALI culture plates were centrifuged at 350 × *g* for 5 min at room temperature to allow the T cells to settle, and supernatants from both sides were discarded. The apical side of the control wells infected with the free virus was washed twice with 200 µL medium; the last wash was collected, and it refers to the day 0 “progeny” in the progeny titrations. The ALI cultures were incubated at 37°C in 500 µL ALI culture medium containing 50 units/mL IL-2 added to the basolateral side, and the inserts were transferred to fresh medium every 2–3 days. In order to retain the T cells on the basolateral side, T cells were pelleted from the “old” basolateral medium and transferred to the fresh culture medium. The cultures were recorded daily by imaging the GFP and transmission light signals using Molecular Devices high-throughput microscope (IXM-XL). Ciliated cell beating was visually inspected under the microscope and confirmed for all inserts for the entire duration of the experiment. The presence of a tight ALI monolayer was confirmed by the absence of media leakage to the apical side of the inserts. Only at 21 dpi, the positive control infection with cell-free virus began to show media leakage to the apical side. On day 5 post-T cell addition, GFP-positive epithelial cells were observed, and apical washes with 210 µL medium added to the apical side for 20 min at 37°C were collected at 2- or 3-day intervals for virus progeny titrations.

For the titration of progeny virus in the apical washes, about 5,000 HeLa cells were seeded on 96-well imaging plates and incubated over two nights in complete DMEM. Serial dilutions of the apical washes (three technical replicates) in complete DMEM were used to infect the HeLa cells, and GFP signal was scored at 24 hpi using Molecular Devices high-throughput microscope IXM-C in widefield mode with a 10× objective and six sites per well. Image analyses were carried out as described below. Virus progeny titers were estimated from wells that displayed 20%–40% infection efficiencies, and one GFP-positive cell was taken as one infectious unit. The approximate infectious unit titers per milliliter were calculated by multiplying the fraction of infected cells/well with the estimated number of cells per well at the time of infection (22,000 cells) and taking the dilution factor into account. The infectious titers were normalized to the day 0 values.

### Image analysis

Microscopy images were analyzed and quantified using CellProfiler (version 4.2.1; http://cellprofiler.org) and KNIME (version 3.7.1; https://www.knime.com/knime-analytics-platform) software and custom-programmed pipelines. The nuclei were segmented using the DAPI channel, and the mean GFP or Alexa Fluor 594 intensity on the nuclei mask was measured. The thresholding for infection scoring was based on the GFP or Alexa Fluor 594 99.5% cut-off intensities derived from the non-infected controls. KNIME pipelines were used for determining the number of infected cells per well. GraphPad Prism was used for visual representation of the data.

## Data Availability

The raw data used to create the figures in the article, as well as the CellProfiler pipelines and result Microsoft Excel files, are deposited at Zenodo.org (10.5281/zenodo.15050840). Due to the large size of the imaging data linked to Fig. 3 only result summary Microsoft Excel files are included in the deposited files, but the original raw imaging data are available from the authors upon request.
